# Potassium (2,2′-bipyridine-κ^2^ *N*,*N*′)bis­(carbonato-κ^2^ *O*,*O*′)cobaltate(III) dihydrate

**DOI:** 10.1107/S1600536810037463

**Published:** 2010-09-30

**Authors:** Jian-Fei Wang, Jian-Li Lin

**Affiliations:** a State Key Lab. Base of Novel Functional Materials and Preparation Science, Center of Applied Solid State Chemistry Research, Ningbo University, Ningbo, Zhejiang, 315211, People’s Republic of China

## Abstract

In the title compound, K[Co(CO_3_)_2_(C_10_H_8_N_2_)]·2H_2_O, the Co(III) atom is coordinated by two bipyridine N atoms and four O atoms from two bidentate chelating carbonate anions, and thus adopts a distorted octa­hedral N_2_O_4_ environment. The [Co(bipy)(CO_3_)_2_]^−^ (bipy is 2,2′-bipyridine) ­units are stacked along [100] *via* π–π stacking inter­actions, with inter­planar distances between the bipyridine rings of 3.36 (4) and 3.44 (6) Å, forming chains. Classical O—H⋯O hydrogen-bonding inter­actions link the chains, forming channels along (100) in which the K^+^ ions reside and leading to a three-dimensional supra­molecular architecture.

## Related literature

For general background to Co(III) complexes, see: Baca *et al.* (2005 ▶); Niederhoffer *et al.* (1982 ▶); Ma *et al.* (2008 ▶). For a related structure, see: Lv *et al.* (2007 ▶).
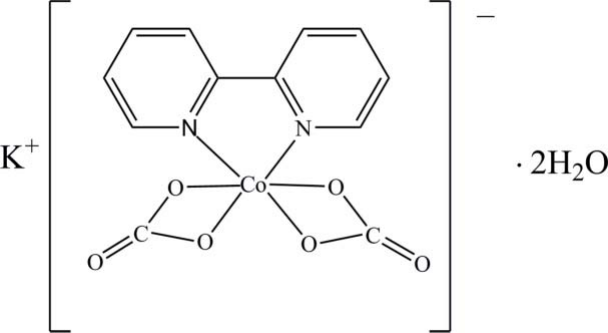


## Experimental

### 

#### Crystal data

K[Co(CO_3_)_2_(C_10_H_8_N_2_)]·2H_2_O
*M* *_r_* = 410.27
Monoclinic, 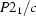

*a* = 7.4138 (15) Å
*b* = 14.064 (3) Å
*c* = 15.392 (4) Å
β = 113.80 (3)°
*V* = 1468.4 (7) Å^3^
*Z* = 4
Mo *K*α radiation
μ = 1.50 mm^−1^
*T* = 295 K
0.58 × 0.18 × 0.17 mm

#### Data collection

Rigaku R-AXIS RAPID diffractometer
Absorption correction: multi-scan (*ABSCOR*; Higashi, 1995 ▶) *T*_min_ = 0.731, *T*_max_ = 0.775
13677 measured reflections
3249 independent reflections
2792 reflections with *I* > 2σ(*I*)
*R*_int_ = 0.023

#### Refinement

*R*[*F* ^2^ > 2σ(*F* ^2^)] = 0.030
*wR*(*F* ^2^) = 0.078
*S* = 1.04
3249 reflections
217 parameters
H-atom parameters constrained
Δρ_max_ = 0.36 e Å^−3^
Δρ_min_ = −0.39 e Å^−3^

### 

Data collection: *RAPID-AUTO* (Rigaku, 1998 ▶); cell refinement: *RAPID-AUTO*; data reduction: *CrystalStructure* (Rigaku/MSC, 2004 ▶); program(s) used to solve structure: *SHELXS97* (Sheldrick, 2008 ▶); program(s) used to refine structure: *SHELXL97* (Sheldrick, 2008 ▶); molecular graphics: *SHELXTL* (Sheldrick, 2008 ▶); software used to prepare material for publication: *SHELXTL*.

## Supplementary Material

Crystal structure: contains datablocks global, I. DOI: 10.1107/S1600536810037463/rk2228sup1.cif

Structure factors: contains datablocks I. DOI: 10.1107/S1600536810037463/rk2228Isup2.hkl

Additional supplementary materials:  crystallographic information; 3D view; checkCIF report

## Figures and Tables

**Table 1:** Hydrogen-bond geometry (Å, °)

*D*—H⋯*A*	*D*—H	H⋯*A*	*D*⋯*A*	*D*—H⋯*A*
O7—H7*A*⋯O8^i^	0.86	2.11	2.934 (3)	161
O7—H7*B*⋯O3^ii^	0.85	2.06	2.903 (3)	170
O8—H8*A*⋯O4^iii^	0.85	2.06	2.885 (3)	162
O8—H8*B*⋯O1^iv^	0.85	2.17	2.988 (3)	162

Symmetry codes: (i) 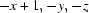; (ii) 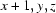; (iii) 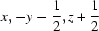; (iv) 
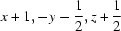
.

**Fig. 1.**
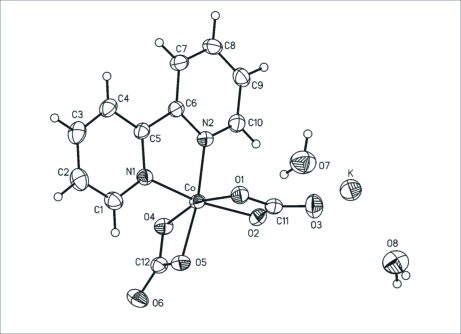
The content of asymmetric unit showing the atomic numbering scheme. The displacement ellipsoids are presented at 45% probability level. The H atoms are drawn as a small spheres of arbitrary radius.

**Fig. 2.**
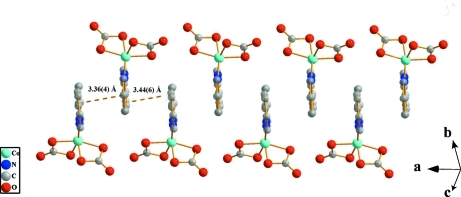
A portion of the crystal packing viewed along axis a and showing the polymeric chains composed from the Co3+ ions and 2,2'–bipy ligands. The π···π stacking interactions are indicated by dashed lines. K+ ions and lattice water molecules were omitted for clarity.

**Fig. 3.**
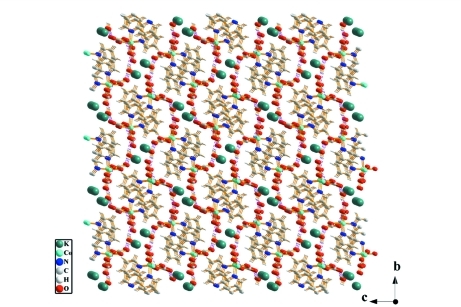
A portion of the crystal packing and showing O—H···O hydrogen bonds as dashed lines.

## supplementary crystallographic information

### Comment

Over the past dacades, the Co(III) complexes with carbonate anions and 2,2'-bipyridine (*bipy*) have done vast efforts due to their interesting coordination, potential applications in magnetism, electronics, *etc*. (Baca *et al.*, 2005). Many this kind of complexes which have been reported are coordinated by two *bipy* molecules and one carbonate anion, leading to [Co(*bipy*)_2_(CO_3_)]^+^ ion (Niederhoffer *et al.*, 1982; Ma *et al.*, 2008). In this contribution, we report the title compound with [Co(*bipy*)(CO_3_)_2_]^-^ anion which coordinated by one *bipy* molecule and two carbonate anions.

The asymmetric unit of this title compound, K[Co(*bipy*)(CO_3_)_2_]^.^2H_2_O, contains one Co(III) ion, one K(I) ion, one *bipy*, two carbonate anions and two lattice water molecules (Fig. 1). The Co(III) atoms are each coordinated by two bipyridine nitrogen atoms and four oxygen atoms from two bidentate chelating carbonate anions, and thus adopts a distorted octahedral N_2_O_4_ environment. The equatorial plane is defined by two nitrogen atoms from one *bipy* ligand and two chelated oxygen atoms from a carbonate ion, while the other two oxygen atoms of the second carbonate ion occupy the axial positions. The Co–O distance of 1.8950 (14)–1.9170 (15)Å, are shorter than those to the nitrogen atoms (Co–N = 1.9219 (17)–1.9325 (19)Å) which are similar to the literatures (Lv *et al.*, 2007). The *trans*– and *cisoid* angles fall in the regions 68.98 (6)–101.89 (7)° and 162.02 (6)–169.86 (7)°, respectively, exhibiting small deviation from the corresponding values for a regular geometry and the above bonding values about the Co atoms are all within the normal ranges. The K^+^ cations are each surrounded by seven O atoms belonging to three water molecules and four carboxylate groups with usual K–O contact distances.

Along [1 0 0] direction, the [Co(*bipy*)(CO_3_)_2_]^-^ unit are stacked through π···π stacking interactions (interplanar distances between *bipy* rings 3.36 (4) and 3.44 (6) Å) to form the one–dimensional chain metallacycle (Fig. 2). The hydrogen bonding O—H···O interactions interlink the chains to form the long–tunnel channels in (1 0 0), which accommodate the K^+^ ions, and thus lead into three–dimensional supramolecular architecture (Fig. 3).

### Experimental

Addition of 5 drops 1.0 *M* NaOH to an aqueous solution of CoCl_2_^.^6H_2_O (0.238 g, 1.0 mmol) in 5.0 ml H_2_O produced a wine red precipitate, which was separated by centrifugation and washed with distilled water for 5 times. The precipitate was then transferred into an aqueous solution of *bipy* (2,2'–bipyridine) (0.156 g, 1.0 mmol) in 10.0 ml CH_3_OH, and dropped 1*M* K_2_CO_3_ to form a clear brownish red solution (pH = 12.6) under stirring. After slow evaporation of the solution black crystals afforded after several weeks at room temperature.

### Refinement

The H atoms bonded to C atoms were placed in geometrically calculated positions with C—H distances 0.93Å and were refined using a riding model with *U*_iso_(H) = 1.2*U*_eq_(C). The H atoms attached to O atoms were found in a difference Fourier synthesis and were refined using a riding model with the O—H distances fixed as initially found and with *U*_iso_(H) = 1.2*U*_eq_(O).

### Crystal data

**Table:**

K[Co(CO_3_)_2_(C_10_H_8_N_2_)]·2H_2_O	*F*(000) = 832
*M**_r_* = 410.27	*D*_x_ = 1.856 Mg m^−^^3^
Monoclinic, *P*2_1_/*c*	Mo *K*α radiation, λ = 0.71073 Å
Hall symbol: -P 2ybc	Cell parameters from 11721 reflections
*a* = 7.4138 (15) Å	θ = 3.0–27.5°
*b* = 14.064 (3) Å	µ = 1.50 mm^−^^1^
*c* = 15.392 (4) Å	*T* = 295 K
β = 113.80 (3)°	Chip, black
*V* = 1468.4 (7) Å^3^	0.58 × 0.18 × 0.17 mm
*Z* = 4	

### Data collection

**Table:**

Rigaku R-AXIS RAPID diffractometer	3249 independent reflections
Radiation source: fine-focus sealed tube	2792 reflections with *I* > 2σ(*I*)
graphite	*R*_int_ = 0.023
Detector resolution: 0 pixels mm^-1^	θ_max_ = 27.5°, θ_min_ = 3.0°
ω–scan	*h* = −8→9
Absorption correction: multi-scan (*ABSCOR*; Higashi, 1995)	*k* = −18→18
*T*_min_ = 0.731, *T*_max_ = 0.775	*l* = −19→19
13677 measured reflections	

### Refinement

**Table:**

Refinement on *F*^2^	Primary atom site location: structure-invariant direct methods
Least-squares matrix: full	Secondary atom site location: difference Fourier map
*R*[*F*^2^ > 2σ(*F*^2^)] = 0.030	Hydrogen site location: inferred from neighbouring sites
*wR*(*F*^2^) = 0.078	H-atom parameters constrained
*S* = 1.03	*w* = 1/[σ^2^(*F*_o_^2^) + (0.0412*P*)^2^ + 0.8088*P*] where *P* = (*F*_o_^2^ + 2*F*_c_^2^)/3
3249 reflections	(Δ/σ)_max_ = 0.001
217 parameters	Δρ_max_ = 0.36 e Å^−^^3^
0 restraints	Δρ_min_ = −0.39 e Å^−^^3^

### Special details

**Table:**

Geometry. All s.u.'s (except the s.u. in the dihedral angle between two l.s. planes) are estimated using the full covariance matrix. The cell s.u.'s are taken into account individually in the estimation of s.u.'s in distances, angles and torsion angles; correlations between s.u.'s in cell parameters are only used when they are defined by crystal symmetry. An approximate (isotropic) treatment of cell s.u.'s is used for estimating s.u.'s involving l.s. planes.
Refinement. Refinement of *F*^2^ against ALL reflections. The weighted *R*–factor *wR* and goodness of fit *S* are based on *F*^2^, conventional *R*–factors *R* are based on *F*, with *F* set to zero for negative *F*^2^. The threshold expression of *F*^2^ > σ(*F*^2^) is used only for calculating *R*–factors(gt) *etc*. and is not relevant to the choice of reflections for refinement. *R*–factors based on *F*^2^ are statistically about twice as large as those based on *F*, and *R*–factors based on ALL data will be even larger.

### Fractional atomic coordinates and isotropic or equivalent isotropic displacement parameters (Å^2^)

**Table:**

	*x*	*y*	*z*	*U*_iso_*/*U*_eq_	
Co	−0.15263 (4)	−0.183676 (18)	−0.376007 (18)	0.02336 (9)	
K	0.17639 (8)	−0.06463 (4)	−0.05791 (4)	0.04510 (15)	
O1	−0.3391 (2)	−0.15333 (10)	−0.32390 (10)	0.0307 (3)	
O2	−0.0295 (2)	−0.17746 (10)	−0.24051 (10)	0.0297 (3)	
O3	−0.2175 (3)	−0.13028 (13)	−0.16521 (12)	0.0458 (4)	
O4	0.0521 (2)	−0.24608 (10)	−0.40039 (11)	0.0301 (3)	
O5	−0.1835 (2)	−0.31752 (10)	−0.37814 (10)	0.0300 (3)	
O6	0.0328 (2)	−0.40547 (11)	−0.41275 (12)	0.0412 (4)	
O7	0.4913 (3)	−0.02509 (15)	−0.12247 (15)	0.0601 (5)	
H7A	0.5011	0.0352	−0.1276	0.090*	
H7B	0.5648	−0.0574	−0.1420	0.090*	
O8	0.4066 (3)	−0.17732 (14)	0.09211 (15)	0.0536 (5)	
H8A	0.3177	−0.2011	0.1073	0.080*	
H8B	0.5008	−0.2166	0.1180	0.080*	
N1	−0.3278 (2)	−0.16972 (11)	−0.50825 (12)	0.0262 (3)	
N2	−0.0978 (2)	−0.05200 (11)	−0.38840 (12)	0.0256 (3)	
C1	−0.4355 (3)	−0.23832 (15)	−0.56647 (16)	0.0333 (5)	
H1	−0.4197	−0.3008	−0.5448	0.040*	
C2	−0.5694 (3)	−0.21909 (18)	−0.65784 (17)	0.0405 (5)	
H2	−0.6425	−0.2679	−0.6970	0.049*	
C3	−0.5931 (3)	−0.12702 (18)	−0.68993 (16)	0.0391 (5)	
H3	−0.6847	−0.1126	−0.7507	0.047*	
C4	−0.4802 (3)	−0.05601 (16)	−0.63147 (15)	0.0338 (5)	
H4	−0.4929	0.0066	−0.6526	0.041*	
C5	−0.3477 (3)	−0.07938 (14)	−0.54072 (14)	0.0257 (4)	
C6	−0.2142 (3)	−0.01164 (14)	−0.47241 (14)	0.0253 (4)	
C7	−0.1996 (3)	0.08361 (15)	−0.48953 (16)	0.0333 (5)	
H7	−0.2809	0.1103	−0.5474	0.040*	
C8	−0.0630 (4)	0.13897 (15)	−0.41985 (17)	0.0375 (5)	
H8	−0.0521	0.2035	−0.4299	0.045*	
C9	0.0568 (3)	0.09727 (16)	−0.33514 (16)	0.0361 (5)	
H9	0.1503	0.1332	−0.2874	0.043*	
C10	0.0361 (3)	0.00167 (15)	−0.32208 (15)	0.0324 (5)	
H10	0.1184	−0.0264	−0.2651	0.039*	
C11	−0.1975 (3)	−0.15262 (14)	−0.23757 (15)	0.0295 (4)	
C12	−0.0283 (3)	−0.32881 (14)	−0.39785 (14)	0.0283 (4)	

### Atomic displacement parameters (Å^2^)

**Table:**

	*U*^11^	*U*^22^	*U*^33^	*U*^12^	*U*^13^	*U*^23^
Co	0.02390 (15)	0.02063 (14)	0.02346 (14)	0.00084 (9)	0.00739 (11)	0.00296 (10)
K	0.0431 (3)	0.0460 (3)	0.0428 (3)	0.0059 (2)	0.0137 (2)	0.0018 (2)
O1	0.0291 (8)	0.0311 (7)	0.0311 (7)	0.0021 (6)	0.0113 (7)	0.0006 (6)
O2	0.0290 (8)	0.0323 (8)	0.0245 (7)	0.0021 (5)	0.0073 (6)	0.0051 (6)
O3	0.0531 (10)	0.0532 (11)	0.0372 (9)	−0.0079 (8)	0.0246 (8)	−0.0093 (8)
O4	0.0288 (7)	0.0273 (7)	0.0347 (8)	0.0002 (5)	0.0135 (7)	0.0026 (6)
O5	0.0303 (8)	0.0244 (7)	0.0346 (8)	−0.0014 (5)	0.0123 (7)	0.0045 (6)
O6	0.0435 (9)	0.0286 (8)	0.0488 (9)	0.0056 (7)	0.0157 (8)	−0.0044 (7)
O7	0.0633 (13)	0.0528 (12)	0.0708 (13)	0.0002 (9)	0.0338 (11)	0.0044 (10)
O8	0.0389 (10)	0.0588 (12)	0.0660 (13)	0.0165 (8)	0.0242 (10)	0.0103 (9)
N1	0.0266 (9)	0.0253 (8)	0.0257 (8)	0.0019 (6)	0.0094 (7)	−0.0001 (6)
N2	0.0277 (8)	0.0230 (8)	0.0261 (8)	−0.0002 (6)	0.0108 (7)	0.0011 (6)
C1	0.0328 (11)	0.0293 (11)	0.0337 (11)	−0.0019 (8)	0.0091 (10)	−0.0031 (8)
C2	0.0347 (12)	0.0458 (13)	0.0343 (11)	−0.0046 (10)	0.0068 (10)	−0.0098 (10)
C3	0.0293 (11)	0.0542 (15)	0.0262 (10)	0.0076 (10)	0.0034 (9)	0.0017 (10)
C4	0.0348 (12)	0.0380 (12)	0.0276 (10)	0.0095 (9)	0.0117 (9)	0.0075 (8)
C5	0.0254 (10)	0.0283 (10)	0.0237 (9)	0.0058 (7)	0.0103 (8)	0.0031 (7)
C6	0.0265 (10)	0.0258 (9)	0.0266 (9)	0.0039 (7)	0.0137 (8)	0.0021 (7)
C7	0.0415 (12)	0.0268 (10)	0.0343 (11)	0.0052 (8)	0.0183 (10)	0.0065 (8)
C8	0.0525 (14)	0.0224 (10)	0.0451 (13)	−0.0022 (9)	0.0275 (11)	0.0007 (9)
C9	0.0464 (13)	0.0306 (11)	0.0363 (11)	−0.0102 (9)	0.0218 (11)	−0.0086 (9)
C10	0.0369 (12)	0.0322 (11)	0.0273 (10)	−0.0041 (8)	0.0120 (9)	−0.0011 (8)
C11	0.0342 (11)	0.0238 (9)	0.0309 (10)	−0.0042 (8)	0.0135 (9)	0.0009 (8)
C12	0.0279 (10)	0.0265 (10)	0.0253 (10)	0.0024 (7)	0.0055 (9)	0.0020 (7)

### Geometric parameters (Å, °)

**Table:**

Co—O5	1.8950 (14)	N2—C10	1.334 (3)
Co—O1	1.9058 (15)	N2—C6	1.356 (3)
Co—O2	1.9115 (16)	C1—C2	1.382 (3)
Co—O4	1.9170 (15)	C1—H1	0.9300
Co—N2	1.9219 (17)	C2—C3	1.372 (4)
Co—N1	1.9325 (19)	C2—H2	0.9300
Co—C12	2.319 (2)	C3—C4	1.378 (3)
Co—C11	2.327 (2)	C3—H3	0.9300
O1—C11	1.320 (3)	C4—C5	1.385 (3)
O2—C11	1.312 (3)	C4—H4	0.9300
O3—C11	1.222 (3)	C5—C6	1.467 (3)
O4—C12	1.315 (2)	C6—C7	1.378 (3)
O5—C12	1.312 (3)	C7—C8	1.380 (3)
O6—C12	1.226 (3)	C7—H7	0.9300
O7—H7A	0.8570	C8—C9	1.378 (3)
O7—H7B	0.8517	C8—H8	0.9300
O8—H8A	0.8513	C9—C10	1.377 (3)
O8—H8B	0.8529	C9—H9	0.9300
N1—C1	1.339 (3)	C10—H10	0.9300
N1—C5	1.351 (3)		
			
O5—Co—O1	97.29 (6)	N1—C1—H1	119.0
O5—Co—O2	93.75 (6)	C2—C1—H1	119.0
O1—Co—O2	68.83 (7)	C3—C2—C1	119.1 (2)
O5—Co—O4	68.98 (6)	C3—C2—H2	120.5
O1—Co—O4	162.02 (6)	C1—C2—H2	120.5
O2—Co—O4	99.62 (7)	C2—C3—C4	119.6 (2)
O5—Co—N2	169.86 (7)	C2—C3—H3	120.2
O1—Co—N2	92.53 (7)	C4—C3—H3	120.2
O2—Co—N2	92.13 (7)	C3—C4—C5	118.9 (2)
O4—Co—N2	101.89 (7)	C3—C4—H4	120.5
O5—Co—N1	93.30 (6)	C5—C4—H4	120.5
O1—Co—N1	97.31 (7)	N1—C5—C4	121.56 (19)
O2—Co—N1	165.15 (7)	N1—C5—C6	113.83 (17)
O4—Co—N1	95.10 (7)	C4—C5—C6	124.59 (19)
N2—Co—N1	82.92 (7)	N2—C6—C7	121.37 (19)
O5—Co—C12	34.46 (7)	N2—C6—C5	113.41 (17)
O1—Co—C12	131.03 (7)	C7—C6—C5	125.21 (18)
O2—Co—C12	98.95 (7)	C6—C7—C8	119.3 (2)
O4—Co—C12	34.54 (7)	C6—C7—H7	120.3
N2—Co—C12	136.17 (8)	C8—C7—H7	120.3
N1—Co—C12	94.25 (7)	C9—C8—C7	119.1 (2)
O5—Co—C11	98.13 (7)	C9—C8—H8	120.5
O1—Co—C11	34.56 (7)	C7—C8—H8	120.5
O2—Co—C11	34.32 (7)	C10—C9—C8	119.1 (2)
O4—Co—C11	133.02 (7)	C10—C9—H9	120.4
N2—Co—C11	91.38 (7)	C8—C9—H9	120.4
N1—Co—C11	131.44 (8)	N2—C10—C9	122.2 (2)
C12—Co—C11	120.66 (7)	N2—C10—H10	118.9
C11—O1—Co	90.44 (12)	C9—C10—H10	118.9
C11—O2—Co	90.44 (12)	O3—C11—O2	124.5 (2)
C12—O4—Co	89.72 (12)	O3—C11—O1	125.4 (2)
C12—O5—Co	90.76 (11)	O2—C11—O1	110.12 (18)
H7A—O7—H7B	113.9	O3—C11—Co	175.80 (17)
H8A—O8—H8B	101.3	O2—C11—Co	55.24 (10)
C1—N1—C5	118.89 (18)	O1—C11—Co	54.99 (10)
C1—N1—Co	126.68 (14)	O6—C12—O5	125.04 (19)
C5—N1—Co	114.31 (13)	O6—C12—O4	124.5 (2)
C10—N2—C6	118.92 (18)	O5—C12—O4	110.48 (17)
C10—N2—Co	126.24 (14)	O6—C12—Co	177.71 (17)
C6—N2—Co	114.80 (13)	O5—C12—Co	54.78 (9)
N1—C1—C2	122.0 (2)	O4—C12—Co	55.74 (10)

### Hydrogen-bond geometry (Å, °)

**Table:**

*D*—H···*A*	*D*—H	H···*A*	*D*···*A*	*D*—H···*A*
O7—H7A···O8^i^	0.86	2.11	2.934 (3)	161
O7—H7B···O3^ii^	0.85	2.06	2.903 (3)	170
O8—H8A···O4^iii^	0.85	2.06	2.885 (3)	162
O8—H8B···O1^iv^	0.85	2.17	2.988 (3)	162

Symmetry codes: (i) −*x*+1, −*y*, −*z*; (ii) *x*+1, *y*, *z*; (iii) *x*, −*y*−1/2, *z*+1/2; (iv) *x*+1, −*y*−1/2, *z*+1/2.

## References

[bb1] Baca, S. G., Filippova, I. G., Ambrus, C., Gdaniec, M., Simonov, Yu. A., Gerbeleu, N., Gherco, O. A. & Decurtins, S. (2005). *Eur. J. Inorg. Chem.* pp. 3118–3130.

[bb2] Higashi, T. (1995). *ABSCOR* Rigaku Corporation, Tokyo, Japan.

[bb3] Lv, Y.-X., Ling, Y., Li, H. & Zhang, L. (2007). *Acta Cryst.* E**63**, m1906–m1907.

[bb4] Ma, P.-T., Wang, Y.-X., Zhang, G.-Q. & Li, M.-X. (2008). *Acta Cryst.* E**64**, m14. 10.1107/S1600536807062551 PMC2914907 21200495

[bb5] Niederhoffer, E. C., Martell, A. E., Rudolf, P. & Clearfield, A. (1982). *Inorg. Chem.* pp. 3734–3741.

[bb6] Rigaku (1998). *RAPID-AUTO* Rigaku Corporation, Tokyo, Japan.

[bb7] Rigaku/MSC (2004). *CrystalStructure* Rigaku/MSC Inc., The Woodlands, Texas, USA.

[bb8] Sheldrick, G. M. (2008). *Acta Cryst.* A**64**, 112–122. 10.1107/S0108767307043930 18156677

